# Rapid Onset of Weight Gain and Liver Dysfunction Successfully Treated With Nutrition and Exercise

**DOI:** 10.7759/cureus.16530

**Published:** 2021-07-21

**Authors:** Hiroteru Kamimura, Masakazu Sano, Takanori Tsujimura, Yasunaga Takeda, Yuko Komoro, Junji Yokoyama, Shuji Terai

**Affiliations:** 1 Gastroenterology and Hepatology, Niigata University, Niigata, JPN; 2 Neurological Surgery, Niigata University, Niigata, JPN; 3 Oral Medicine, Niigata University, Niigata, JPN; 4 Nutrition, Niigata University, Niigata, JPN

**Keywords:** exercise, liver dysfunction, inactivity, nutrition, nafld

## Abstract

Physical inactivity is one of the causes of most metabolic syndromes. The incidence of metabolic syndrome is expected to increase in the near future because of the reduced opportunities for exercise caused by COVID-19. Non-alcoholic fatty liver disease (NAFLD) is currently the most common cause of chronic liver disease. Changes in diet and lifestyle have led to a dramatic increase in the prevalence of NAFLD in the world. NAFLD is characterized by excessive triglyceride (TG) accumulation in the hepatocytes due to both increased inflow of free fatty acids and de novo hepatic lipogenesis. Thus far, no study quantitatively assessed the liver fat deposition after a rapid decline in physical activity. Herein, we describe a case of a 17-year-old Japanese boy with severe fat infiltration of the liver, due to a rapid decline in physical activity, treated at our facility. Our rehabilitation and nutritional support teams administered appropriate exercise and nutrition support to reduce weight and improve liver dysfunction. Our findings support dietary changes and exercise therapy to manage such cases.

## Introduction

Non-alcoholic fatty liver disease (NAFLD) is characterized by excessive triglyceride (TG) accumulation in the hepatocytes due to both increased inflow of free fatty acids and de novo hepatic lipogenesis. NAFLD is the most common chronic liver disease in developed countries, and its incidence is increasing, warranting the attention of primary care physicians. One-ﬁfth to one-quarter of adults in the developed world suffer from NAFLD [1], a systemic condition associated with physical inactivity and obesity, also known as a metabolic syndrome. The disease is considered a phenotype of metabolic syndrome associated with insulin resistance in the liver and manifests as two clinical entities: non-alcoholic fatty liver (NAFL) and non-alcoholic steatohepatitis (NASH) [2]. Although several randomized controlled trials (RCTs) involving patient populations with long-standing obesity and metabolic syndrome have been conducted [3], there are only a few reports regarding the degree of liver fat deposition following a rapid decline in physical activity in individuals without metabolic syndrome. The gold standard for the diagnosis is liver biopsy, which helps assess obesity, inflammation, and hepatocellular ballooning to distinguish between the NAFL and NASH [4]. Furthermore, no study has so far quantitatively assessed fatty deposition over time using CT, controlled attenuation parameter (CAP) obtained from abdominal USG, or bioelectrical impedance analysis (BIA) after a rapid decline in physical activity.

Here, we report a case of a male, high-school athlete who was previously engaged in athletic activities but developed a rapidly progressive liver dysfunction from a fatty liver caused by the inactivity while awaiting chemotherapy for a brain tumor.

This case of a rapid decline in physical activity depends on medical conditions, but our findings support dietary changes and exercise therapy to manage such cases including reduced opportunities for exercise caused by COVID-19.

## Case presentation

**Figure 1:**
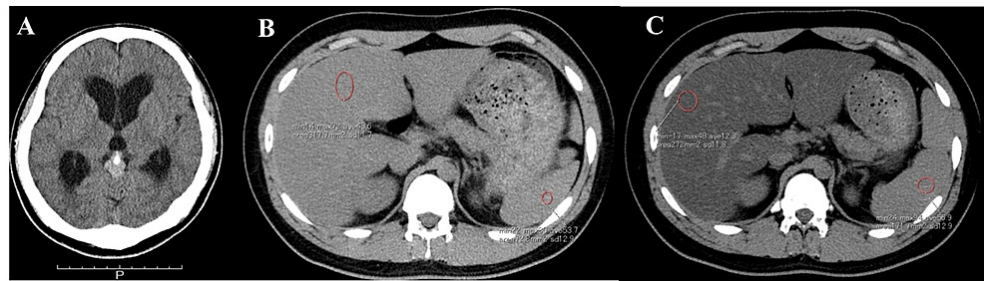
Brain and abdominal CT. 1A. Brain CT: Brain tumor with calcification in the pineal region, with narrowing of the midbrain aqueduct and third and lateral ventricle enlargement. 1B. Initial abdominal CT: CT revealed a slightly lower attenuation of the liver/spleen (L/S) ratio of 0.81. 1C. Abdominal CT at the time of consultation: CT showed that the L/S ratio was extremely decreased to 0.21, 41 days later, following the period of inactivity.

A 17-year-old male high-school student with no history of illness or liver disease had persistent headaches for approximately three weeks and visited his primary care physician, who diagnosed hydrocephalus and possible intracranial hypertension and referred him to our hospital. He was a highly competitive school baseball player with no history of alcohol abuse. He was found to have a brain tumor with calcification in the pineal region, with narrowing of the midbrain aqueduct and the third and lateral ventricle enlargement (Figure 1A). He was managed with an emergency endoscopic third ventriculostomy, biopsy, and reservoir implantation. He was discharged from the hospital while awaiting biopsy results, and the final diagnosis was a pineal germ cell tumor. Between biopsy and chemo-radiotherapy, the patient was waiting for scheduled hospitalization. There was a 41-day waiting period at home, during which the patient had discontinued his sporting activities but maintained the same food intake.

At the time of his second admission for chemotherapy and radiotherapy, laboratory examination showed severe hepatic dysfunction with aspartate aminotransferase (AST) and alanine aminotransferase (ALT) levels of 85 (normal, 8-38) and 195 (normal, 4-44) U/L respectively, which had been within the normal range 41 days ago (Table 1). The attending neurosurgeons consulted our hepatology department. On examination, his height and weight were 177 cm and 97 kg, respectively. He had gained 14 kg since his first admission to the hospital 41 days ago, demonstrating a significant increase in his BMI from 26 to 31 kg/m2.

**Table 1:** Laboratory data on admission. Alb: Albumin; ALP: Alkaline phosphatase; ALT: Alanine aminotransferase; ANA: Antinuclear antibody; AMA: Antimitochondrial antibody; AMA-M2: Antimitochondrial M2 antibody; LKM1: Antiliver/Kidney microsome type 1 antibody; APTT: Activated partial thromboplastin time; AST: Aspartate aminotransferase; BUN: Blood urea nitrogen; CBC: Complete blood count; CHE: Cholinesterase; Cr: Creatinine; CRP: C-reactive protein; D-bil: Direct bilirubin; Hb: Hemoglobin; Ht: Hematocrit; LDH: Lactate dehydrogenase; Plt: Platelets; PT: Prothrombin time; T-bil: Total bilirubin; T-chol: Total cholesterol; TP: Total protein; γ-GT: γ-Glutamyltransferase.

Data on admission					
【CBC】		【Chemistry】			
WBC	7700/μl	T-bil	0.6 mg/dl		
Neut	72.0%	D-bil	0.1 mg/dl	ANA	<40
Eo	3.2%	AST	72 IU/l	AMA	(-)
Baso	0.5%	ALT	173 IU/l	HBsAg	(-)
Mon	5.5%	ALP	346 IU/l	anti-HBs	(-)
Lymph	18.6%	LDH	165 IU/L	anti-HCV	(-)
RBC	482 x 10^4^/μl	CHE	123 IU/l		
Hb	14.8 g/dl	γ-GT	99 IU/l	Total carnitine	76.2 µg/dL
Ht	43.60%	TP	7.9 g/dl	Cu	90 µg/dL
Plt	28 x 10^4^/μl	Alb	3.0 g/dl	Ceruloplasmin	20 mg/dL
		CRP	0.2 mg/dl	TSH	1.07 μIU/m
【Coagulation】		T-Cho	74 mg/dl	FT3	2.8 pg/m
PT	108%	TG	47 mg/dl	FT4	1.15 ng/ml
APTT	33.6 sec	HbA1c	5.10%	Somatomedin-c (Insulin-like growth factor-1).	302 ng/ml
Fibrinogen	204 mg/dl	BS			
FDP	23.8 μg/ml	BUN	9.1 mg/dl		
		Cr	0.61 mg/dl		
		NH3	77 µg/dl		

Test results for other causes of liver damage, including viral hepatitis and autoimmune hepatitis, were negative. Somatomedin C was found present, and there was no evidence of pituitary hypofunction. Abdominal CT at his first admission revealed a liver/spleen (L/S) ratio of 0.81 with fatty deposits; however, CT at the second admission 41 days later, following the period of inactivity, showed that the L/S ratio decreased to 0.21 (Figure 1B and C). The scheduled chemotherapy was postponed due to these abnormal liver findings. The CAP measured using USG (FibroScan 502 Touch devices equipped with XL probes [Echosens, Paris, France]) demonstrated marked fatty liver changes at 400 dB/m. The BIA (InBody720, Biospace, Seoul, Korea) revealed a muscle mass and body fat content of 62.3 kg and 28.6 kg, respectively. Since the tumor treatment was a priority, we diagnosed the condition as liver damage secondary to the hepatic fatty deposits without a liver biopsy.

His nutrition and exercise levels were assessed, and he was started on a diet and exercise therapy. Before admission, considering the amount of time the patient exercised, he had expended approximately 5-7 metabolic equivalents (METs) and probably spent approximately 1,500-2,000 kcal/day. Although he had been exercising before the diagnosis, he did not continue exercising during the 41 days before his second admission. The nutritional assessment suggested that he continued to consume 160 g of protein, 153 g of lipid, and 548 g of carbohydrate, resulting in an intake of 4,400 kcal/day. This continued caloric intake in the absence of exercise caused a rapid weight gain.

**Figure 2:**
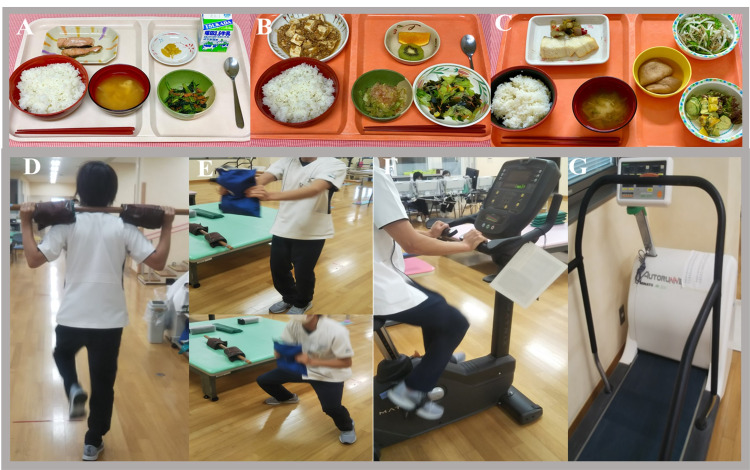
Nutritional and exercise intervention. A-C. Nutritional intervention: 2,400 kcal/day, 75 g of protein (12.5%), 380–400 g of carbohydrate (63%–66%), and 52 g of lipid (20%). 2A. Morning: Rice 275 g, grilled salmon 80 g, greens with sesame sauce, pickles, milk. 2B. Lunch: Rice 275 g, Mapo tofu, seaweed salad, boiled greens, fruit. 2C. Dinner: Rice 275 g, braised Greenland halibut, simmered deep-fried tofu, radish, and pumpkin sweet potato salad. D-G. Exercise intervention: 20 minutes for a total volume of 4–6 metabolic equivalents, starting with a consumption of approximately 200–300 kcal/day during exercise intervention. 2D. The intervention consisted of lower limb strength training with a 6-kg load for 10 min. 2E. Flexion and extension exercises with a 5-kg load. 2F. Using a cycle ergometer (Well Bike BE-260, Fukuda Denshi, Tokyo, Japan) at a power output of 30–100 W. 2G. Running on a treadmill for about 30 minutes.

We provided nutritional guidance that recommended 2,400 kcal/day: 75 g of protein (12.5%), 380-400 g of carbohydrate (63%-66%), and 52 g of lipid (20%) (Figure 2 A, B, C), and simultaneously initiated a daily exercise routine of approximately 20 minutes for a total volume of 4-6 METs, starting with an expenditure of approximately 200-300 kcal/day during the exercise intervention.

The intervention consisted of a lower limb strength training with a 6-kg load for 10 minutes, flexion and extension exercises with a 5-kg load, using a cycle ergometer (Well Bike BE-260, Fukuda Denshi, Tokyo, Japan) at a power output of 30-100 W, and additional in-hospital walking. He continued this exercise therapy for 60 days (Figure 2 D, E, F, G). With this continued rehabilitation, his weight decreased to 87 kg, and his body fat composition was reduced to 26 kg. With these changes, the CAP value for the hepatic fat deposition decreased to 320 dB/m, and the AST and ALT levels also decreased significantly (Table 2). Considering the patient’s age and risks associated with the radiation exposure, we did not perform a CT scan for L/S measurements after the weight loss. After the correction of the liver disorder, he underwent whole-brain, whole-spine irradiation (23.4 Gy/13 Fr), and local boost irradiation (27 Gy/15 Fr) for 28 days, as well as a single course of chemotherapy (carboplatin and etoposide).

**Table 2:** Patient’s clinical course shows chronological changes in the laboratory data, the value for hepatic fat deposition, body composition, and the content for nutritional and exercise intervention. HT: Height; BW: Body weight; AST: Aspartate aminotransferase; ALT: Alanine aminotransferase; GGT: Gamma-glutamyl transferase; ALP: Alkaline phosphatase; HbA1c: Hemoglobin A1c; BS: Blood sugar; TG: Triglyceride; T-chol: Total cholesterol; CT (L/S): Lumbar Spine CT; CAP: Controlled attenuation parameter; METs: Metabolic equivalents.

	Factor	Before admission as an athlete	Consultation to our division	After nutritional and exercise intervention for 60 days
	Day	0	41	101
	HT (cm)	177	177	177
	BW (kg)	83	97	87
	BMI	26	31	28
	AST (U/L)	19	72	28
	ALT (U/L)	27	154	45
	GGT (U/L)	36	99	43
	ALP (U/L)	308	346	570
	HbA1c (%)	5	5.6	5.1
	BS	98	115	97
	TG (mg/dl)	76	141	81
	T-chol (mg/dl)	238	207	206
	Fat quantitative values			
Fat quantitative values	CT（L/S)	0.81	0.2	-
	US CAP (dB/m)	-	400	320
	Body composition			
Body composition	Handgrip	-	45	55
	Soft lean mass (kg)	-	62	58
	Body fat mass (kg)	-	29	26
	Nutrition			
Nutrition	Calorie (kcal)	4400	4400	2400
	Protein (g)	160	160	57
	Lipid (g)	153	153	44
	Carbohydrate (g)	548	548	202
	Exercise			
Exercise	Contents	Aerobic and resistance exercise		Aerobic and resistance exercise
	METs	5-7 METs	No exercise	4-6 METs
	Time	180		20-40
	Kcal	1500-2000		300

**Figure 3:**
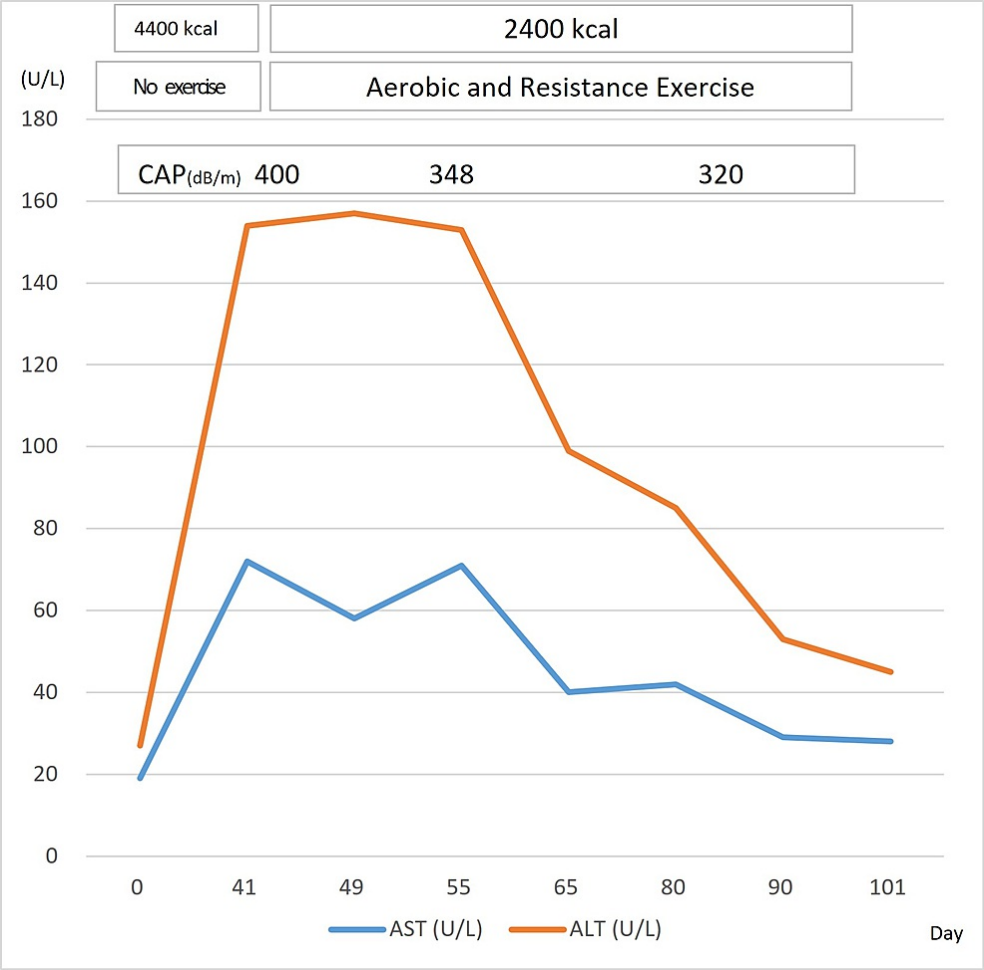
Patient’s transaminase changes during the nutrition and exercise treatment. AST: Aspartate aminotransferase; ALT: Alanine aminotransferase; CAP: Controlled attenuation parameter.

Following discharge from the hospital, the patient returned to his athletic activities, and his weight decreased to 83 kg after two months. His liver function remains normal, and brain MRI showed no tumor recurrence at six months (Figure 3).

## Discussion

This case of rapid decline in physical activity depends on medical conditions, but our findings support dietary changes and exercise therapy to manage such cases including reduced opportunities for exercise caused by COVID-19.

Owing to the rapid decline in physical activity of the general population resulting from the global scale lockdown due to the COVID-19 pandemic and prolonged restriction of movement with the resultant increase in telecommuting, there are concerns regarding various components of metabolic syndrome, muscle weakness, and other health problems [5]. Moreover, people who previously engaged in physical activity, including commuting to work, may rapidly develop inactivity-induced fatty liver [6]. However, this cannot be assessed over a short time without continuous measurements.

Previous studies have reported a metabolic syndrome in retired athletes with no mention of NAFLD [7]. However, there are no similar reports concerning a sudden onset of inactivity in active young individuals.

In 2010, three RCTs were published on histologically proven NASH [8,9,10]. In all three trials, calorie restriction and aerobic exercise improved the histological findings of the liver together with the weight loss in patients with obesity and NAFLD. In our study, a young male athlete who ceased regular physical activity developed fatty liver, but his condition improved with nutritional guidance and exercise intervention during a two-month hospital stay.

Diet and exercise therapy-based weight loss studies have revealed the specific weight loss goals that are necessary to improve the prognosis of NAFLD/NASH. For instance, a weight loss of ≥7% results in improvements in NASH [3], reduces hepatic adipogenesis, inflammatory cell infiltration, hepatocellular ballooning, and improves the NAFLD activity score [11]. A recent study documented improvement in liver tissue, including liver fibrosis, with varying degrees of weight loss, especially with a weight loss of >10%. Thus, a weight loss of 7%-10% or more can lead to histological improvement of the liver. However, the success rates for weight loss of this magnitude are low (18% and 10%, respectively) [9]. Accordingly, lifestyle interventions are challenging to implement given the low rate of target achievement and treatment adherence.

Evidence concerning the degree of weight loss needed, based on hepatic histological assessments and randomized trials involving dietary changes, is scant. However, calorie restriction is essential for dietary interventions to improve NAFLD. Lipids and carbohydrates are often limited to 50%-60% and 20%-25% of the total calories, respectively [12]. The patient in the current case followed this general advice: 2,400 kcal/day comprising 12.5% protein, 63%-66% carbohydrate, and 20% lipids.

Aerobic exercise for 30-60 minutes 3-4 times/week for 4-12 weeks significantly benefits patients with NAFLD and obesity-related complications. Regarding exercise intensity and duration, aerobic exercise for ≥250 minutes/week of moderate to vigorous intensity for 12 weeks effectively improves hepatic adipogenesis in patients with NAFLD. Moreover, continuous aerobic exercise improves fatty liver even in the absence of weight loss [13]. Although the positive effects of aerobic exercise on NAFLD are widely accepted, resistance exercise has recently been reported to be useful [14]. A meta-analysis comparing the aerobic and resistance exercises reported that the resistance exercise was associated with comparable improvement in adipogenesis in the patients with NAFLD, despite a lower total energy expenditure than aerobic exercise. Although these studies did not report histological changes as an endpoint and only provided short-term results, exercise therapy alone may improve hepatic lipolysis and is recommended for the obesity-associated NAFLD/NASH [15]. In our case, the patient engaged in the lower extremity muscle training for approximately 5-10 minutes/day and running, using a cycle ergometer, with a total caloric expenditure of approximately 300 kcal/day.

In this case, to avoid definitive liver biopsy and unnecessary radiation exposure in a young patient with malignant disease, we used USG-based CAP, a method of deep attenuation due to fat deposition, to evaluate the hepatic fat deposition [16]. CAP has been developed to allow highly accurate quantification of the liver fat mass without using invasive procedures. Based on the cut-off values calculated in the previous studies in Europe and the United States, patients with NAFLD and controls fall into four stages: liver fat/cutoff: S0, 0%-10%; S1, 11%-33%, 237.8 dB/m; S2, 34%-62%, 260 dB/m; and S3, 63%-100%, 292.3 dB/m. Preliminary results showed that the proprietary technologies implemented using the ultrasound systems seem more accurate than CAP for grading liver steatosis [17].

In our case, nutritional and exercise interventions improved blood biochemistry test results, such as AST and ALT, along with the improvement in the proportion of body fat and CAP. To identify the patients at risk of progressive NASH noninvasively for clinical trials or treatments, the most predictive and efficient model combined liver stiffness measurement, CAP, and AST and was designated FAST (FibroScan-AST) [18].

For our patient, we compared the findings of dual BIA, which measures visceral fat accumulation, with the CAP measurement. The BIA is a simple, non-invasive assessment tool for body composition [19]. The BIA was measured based on the resistance at 1, 5, 50, 250, and 500 kHz and 1 MHz and reactance at 5, 50, and 250 kHz.

Increased body fat, particularly abdominal visceral fat, is central to the pathogenesis of NAFLD. Identifying a clinically feasible fat assessment method is thus essential. CT and MRI are precise fat assessment methods; however, they are impractical for routine use [20]. According to the previous studies, there is a good correlation between the visceral fat area measured using the BIA and the abdominal CT [21].

Using the BIA, we confirmed the reduction in body weight and body fat mass, and the soft lean mass decreased gradually during the nutrition and exercise intervention.

## Conclusions

In summary, at the peak of the COVID-19 pandemic, we expect an increase in restrictions on outings and exercise opportunities. Our case illustrates that even in adolescent patients, short-term weight gain and fatty liver disease can develop after exercise cessation. However, nutritional and exercise interventions can facilitate appropriate body fat loss and improvements in liver enzyme levels and fatty liver, which can be monitored over time using the CAP and BIA. Dietary changes and exercise therapy can benefit patients in numerous ways and are cost-effective.
